# Large country differences in work outcomes in patients with RA – an analysis in the multinational study COMORA

**DOI:** 10.1186/s13075-017-1421-y

**Published:** 2017-09-29

**Authors:** Antje van der Zee-Neuen, Polina Putrik, Sofia Ramiro, Andras P. Keszei, Ihsane Hmamouchi, Maxime Dougados, Annelies Boonen

**Affiliations:** 1 Department of Internal Medicine, Division of Rheumatology, Maastricht University Medical Centre, Maastricht, The Netherlands; 2 CAPHRI, Maastricht University, Maastricht, The Netherlands; 3 Rheumatology, Leiden University Medical Center, Leiden, Netherlands; 4 Medical Informatics, Uniklinik RWTH Aachen University, Aachen, Germany; 5 Biostatistics Epidemiology LBRCE, Université Mohamed-V Souissi, Rabat, Morocco; 6 Paris Descartes University, Department of Rheumatology - Hôpital Cochin. Assistance Publique-Hôpitaux de Paris INSERM (U1153): Clinical epidemiology and biostatistics, PRES Sorbonne Paris-Cité, Paris, France; 7 Department of Rheumatology, Maastricht University Medical Centre, P/O 5800, NL-6202 AZ Maastricht, The Netherlands

**Keywords:** Multinational, Rheumatoid arthritis, Employment, Absenteeism, Presenteeism

## Abstract

**Background:**

We aimed to explore whether country of residence or specific country characteristics are associated with work outcomes in rheumatoid arthritis (RA).

**Methods:**

Data from the 17 countries participating in the Comorbidities in RA (COMORA) study were used. Work outcomes were measured by the Work Productivity and Activity Impairment Questionnaire, addressing employment (yes/no), absenteeism (percentage of time; 3 categories) and presenteeism (percentage of at-work productivity restrictions; 4 categories). Contribution of country of residence, gross domestic product (GDP), Human Development Index (HDI), unemployment rate, social protection expenditures (SPE) or world region to work outcomes was investigated in adjusted (ordered) logistic regressions.

**Results:**

The patients (n = 2395) were younger than 60 years; mean age 48 (SD 9.2) years, 1972 (84%) female and 1065 (45%) employed. Large country differences were found. Taking the country with the best work outcome as reference, Moroccan patients had the lowest odds of being employed (OR 0.2 (95% CI 0.1; 0.3) vs. Germany) and highest odds of absenteeism (OR 13.2 (3.6; 48.3) vs. Japan). Patients in Taiwan had the highest odds of presenteeism (OR 13.0 (5.5; 30.9) vs. Venezuela). All country indices except SPE were associated with work outcomes. For example, patients in low-GDP countries had lower odds of employment (OR 0.6 (0.5; 0.8)), higher odds of absenteeism (OR 2.8 (2.0; 4.1)), but lower odds of presenteeism (OR 0.5 (0.4; 0.7)) compared to higher-GDP countries.

**Conclusion:**

Substantial differences in work outcomes among patients with RA were observed between countries. Lower economic wealth and human development of countries were associated with worse employment and higher absenteeism, but lower presenteeism.

**Electronic supplementary material:**

The online version of this article (doi:10.1186/s13075-017-1421-y) contains supplementary material, which is available to authorized users.

## Background

The worldwide economic crisis and the aging of the population pose challenges to employment perspectives. On the one hand, there is pressure on individuals to work longer and more efficiently, and on the other hand the number of available jobs is increasingly limited. Persons suffering from a chronic disease such as rheumatoid arthritis (RA) are already at increased risk for adverse work outcomes including restrictions in productivity while at work (presenteeism), sick leave (absenteeism) and eventually work disability or unemployment [1] and therefore are particularly vulnerable to a negative economic climate.

There is increasing evidence that environmental contextual factors such as nature of work or support from colleagues influence work outcome independent of biomedical and personal contextual factors [1, 2]. On this line, limited data suggest that system-level environmental factors, such as the prosperity or development of a country, play a decisive role. Dadoniene et al. observed lower employment rates among patients with RA in Lithuania, a country with an economy in transition, when compared to Norway, one of the wealthiest countries in Europe [3]. Mau et al. reported lower employment in patients with RA residing in the former Eastern compared to Western German states, and attributed this to higher prevailing economic unemployment in former Eastern German states [4]. Chung et al. observed a higher hazard of withdrawal from work due to RA-related work disability in Finland compared to the USA, for similar clinical and sociodemographic characteristics [5], and suggested the more supportive Finnish social security system with favorable disability allowance in case of disease-related work disability partly explained such differences. Finally, the QUEST RA study among 32 countries showed that patients in low-income countries continued to work with high levels of disease activity [6].

Better insight into system-related differences in work outcomes might be important when designing and implementing intervention programs aiming to improve short-term and long-term work outcomes of patients with RA, as the outcome may be strongly dependent on culture or regulations of the country from which participants originate. Moreover, such knowledge is crucial in multinational studies, as stratification by country or (groups of countries) based on a specific characteristic may be essential to avoid bias. As the focus of research on work outcome in rheumatology shifts towards presenteeism (i.e. productivity loss while at work) and sick leave, and as there is increasing evidence that the various work outcomes are differentially influenced by biomedical and contextual factors [7], it is important to address each of these outcomes in addition to employment status.

The Comorbidities in Rheumatoid Arthritis (COMORA) international study collected recent patient-level data from 17 countries across 5 continents including data on work outcomes. These data offer a unique opportunity to explore the impact of country of residence and specific country characteristics on employment, absenteeism and presenteeism. We hypothesized that higher economic wealth and greater human development of a country is associated with higher employment and lower absenteeism and presenteeism.

## Methods

COMORA, an international, cross-sectional multicenter study in 17 countries, collected data in patients diagnosed with RA according to the 1987 American College of Rheumatology classification criteria for RA, who were older than 18 years, and had sufficient command of the questionnaire language. The study protocol was approved by one central and all local institutional ethics committees. Written informed consent was obtained from all subjects. Investigators and patients completed a questionnaire on demographics and self-reported health, including questions on work outcome [8].

### Outcome variables

Work outcome was measured by the Work Productivity Impairment Questionnaire (WPAI) [9–11]. Outcomes comprised current employment status (employed vs. not employed), and in those employed, the percentage of working hours absent (absenteeism) and percentage of at-work productivity restrictions (presenteeism), measured on a numeric rating scale (10 indicating health status completely prevented work) due to health problems during the past 7 days. Persons with long-term disability were therefore considered as not employed, and persons with short-term work disability (sick leave) were considered as employed and included in analyses with absenteeism or presenteeism as the outcome.

### Sociodemographic and clinical characteristics

Sociodemographic and lifestyle information comprised age, gender, highest level of education achieved (primary school, secondary school, university), marital status (single, married, widowed, divorced), smoking status (past, current, never smoked), and weight (kg) and height (cm) from which the body mass index (BMI) was calculated (underweight, BMI < 18.5; normal weight, 18.5 ≥ BMI < 25; overweight, 25 ≥ BMI ≤ 30; and obese, BMI > 30) [12, 13].

Clinical characteristics comprised the modified 8-item Health Assessment Questionnaire (mHAQ) assessing the level of physical function and ranging from 0 to 3 (where 3 = worst function) [14, 15], the 28-joint Disease Activity Scale DAS28 ranging from 0 to (virtually) 10 [16], high rheumatoid factor (RF)/high positive anti-citrullinated protein antibody (ACPA). Physician-confirmed ischemic cardiovascular disease (myocardial infarction, stroke), cancer (colon, skin, lung, breast and uterus in women, prostate in men, and lymphoma), gastrointestinal diseases (diverticulitis, ulcers), infections (hepatitis), lung disease (chronic obstructive pulmonary disease and asthma) and psychiatric disorders (depression) were used to compute the Rheumatic Disease Comorbidity Index (RDCI) [17, 18]. As history of fractures was not collected, these were not included in the RDCI and therefore the score ranged from 0 to 8.

### Country characteristics

Data on socio-economic country indices were extracted from the International Monetary Fund (for gross domestic product (GDP) per capita), the International Labour Organization (for unemployment rates and social protection expenditures (SPE)) and the United Nations Development program (for Human Development Index (HDI)) [19–22]. The GDP per capita is an indicator of economic wealth and is expressed in international dollars (Intl$), to adjust local currency for purchasing power parity (PPP) allowing cross-country comparison [19]. Based on the visual inspection of data, GDP was dichotomized as ≤ Int$20,000 or > Intl$$20,000. Unemployment rate, reflecting economic growth and efficiency, was dichotomized based on the median as ≤ 7.6% or > 7.6% [20]. The HDI reflects a country’s level of development and includes life expectancy, education assessed by years of school attendance and the standard of living (GDP/capita) [21]. HDI was dichotomized based on the United Nations Development Program classification resulting in two categories (moderate versus high and very high). The SPE reflects social expenditure for sickness, maternity, employment injury and disability, and was dichotomized using (a) the percentage SPE of the country’s GDP, dichotomized at the median (<2.15% vs. ≥ 2.15%) and (b) the absolute SPE in international dollars dichotomized at the median (SPE < Intl$801.50 vs. ≥ Intl$801.50) [22]. For all indices we used the most recent year for which the data were available for all countries. For GDP per capita this was 2011, for HDI 2013 and for SPE 2014. Finally, countries were categorized by world region (Africa, Asia, North America, Europe, and Latin America) reflecting socio-cultural coherence. Additional file 1: Table S1 provides an overview of the countries according to the categories of the different indices and the overlap between categories.

### Statistical analysis

Descriptive statistics were used to characterize the total sample. To avoid a strong influence of the age on retirement on the differences between countries in work outcomes, all further analyses were performed on the sample at age ≤ 60 years. Logistic regression was used to understand factors contributing to employment. When absenteeism or presenteeism was the outcome, the sample was limited to those employed and only countries with at least 30 employed persons were considered. A considerable proportion of participants indicated no absence or presenteeism. Based on the model fit (deviance), ordered logistic regression was preferred above zero-inflated regression approaches. Based on inspection and testing for the proportional odds assumption for ordered logistic regression, the categories chosen for absenteeism were 0%, > 0% to <100% and 100%. The categories for presenteeism were 0%, > 0% to 30%, > 30% to 50% and > 50% to 100%. To select the individual covariates associated with each outcome in multivariable models, manual forward selection (cutoff *p* value <0.05) was used. To understand the additional influence of country of residence, “country” was added to the multivariable model as a categorical variable, using the country with the highest employment, or the lowest absenteeism or presenteeism in the raw data as reference. Finally, “country” was replaced by the different country indices. For each model, the fit of models with the different country-related variables was compared to the model without any country variable using log-likelihood ratio tests (with a cutoff of *p* < 0.05 for significance). Statistical models were restricted to complete cases. Interactions between sociodemographic and clinical characteristics with GDP were explored for each work outcome. Analyses were performed using Stata 12 [23].

## Results

Overall, 3920 individuals (with a mean age of 56 years and of whom 81% were women) from 17 countries in 5 world regions comprising *Europe* (i.e. Austria (AT), France (FR), Germany (DE), Hungary (HU), Italy (IT), Netherlands (NL), Spain (ES), United Kingdom (UK)), Latin America (i.e. Uruguay (UR), Venezuela (VE), Argentina (AR)), *Asia* (i.e. Taiwan (TW), Japan (JP), Korea (KR)), Africa (i.e. Morocco (MA), Egypt (EG)) and *North America* (i.e. the United States of America (USA)) were included in COMORA. Clinical and disease characteristics are described in Table 1.

**Table 1:** Characteristics of study sample

Total study sample (n = 3920)									Sample ≤ 60 years, n = 2359			
	Age, mean (SD)	Women, number (percentage)	Level of education, number (percentage)			mHAQ, mean (SD)	DAS28, mean (SD)	Employed, number (percentage)		Employed, number (percentage)	Percent absenteeism, mean (SD)	Percent presenteeism, mean (SD)
			Primary	Secondary	University						n = 993	n = 914
Austria (n = 204)	58.6 (12.7)	167 (81.9)	145 (71.1)	41 (20.1)	18 (8.8)	1.0 (0.6)	3.3 (1.3)	62 (30.4)	n = 105	61 (58.1)	9.4 (24.7)	26.5 (23.5)
Argentina (n = 200)	55.5 (11.7)	175 (87.5)	85 (42.5)	87 (43.5)	28 (14.0)	1.1 (0.6)	4.0 (1.3)	56 (28.0)	n = 133	46 (34.6)	22.4 (36.3)	3136.6 (31.7)
Egypt (n = 308)	47.5 (11.3)	262 (85.1)	190 (61.7)	58 (18.8)	60 (19.5)	1.3 (0.7)	5.3 (1.5)	76 (24.7)	n = 281	74 (26.3)	26.4 (33.9)	31.2 (25.8)
France (n = 411)	58.1 (11.8)	334 (81.3)	112 (27.4)	181 (44.3)	116 (28.4)	0.9 (0.6)	3.1 (1.5)	132 (32.6)	n = 217	116 (53.7)	8.5 (26.1)	20.0 (21.4)
Germany (n = 209)	59.3 (12.2)	152 (72.3)	0 (0.0)	176 (84.2)	33 (15.8)	1.0 (0.7)	3.5 (1.6)	74 (35.5)	n = 113	71 (62.8)	11.2 (30.2)	25.8 (24.5)
Hungary, (n = 201)	59.0 (25.8)	179 (89.1)	38 (18.9)	111 (55.2)	52 (25.9)	1.4 (0.7)	1.4 (0.7)	50 (24.9)	n = 97	43 (44.3)	35.8 (46.0)	22.4 (24.7)
Italy (n = 228)	61.4 (11.7)	176 (77.2)	147 (64.5)	69 (30.3)	12 (5.3)	1.0 (0.6)	3.7 (1.4)	56 (24. 6)	n = 101	50 (49.5)	10.9 (27.8)	31.1 (30.2)
Japan (n = 207)	62.9 (12.7)	168 (81.2)	40 (19.3)	127 (61.4)	40 (19.3)	0.9 (0.6)	3.1 (1.3)	71 (34.3)	n = 72	40 (55.6)	2.6 (9.2)	21.0 (24.2)
Korea (n = 400)	56.3 (11.9)	336 (84.0)	107 (26.8)	194 (48.6)	98 (24.6)	0.9 (0.6)	3.5 (1.4)	118 (29.5)	n = 238	95 (39.9)	2.5 (12.1)	24.7 (23.8)
Morocco (n = 227)	48.3 (12.8)	189 (83.3)	145 (65.6)	41 (18.6)	35 (15.8)	1.5 (0.8)	5.2 (1.6)	34 (15.0)	n = 184	33 (18.2)	43.8 (43.9)	34.0 (22.3)
Netherlands (n = 139)	58.8 (13.7)	91 (65.5)	7 (6.6)	20 (18.9)	79 (74.5)	1.2 (0.6)	2.6 (1.1)	49 (35.3)	n = 70	43 (61.4)	13.8 (30.4)	27.8 (32.2)
Spain (n = 200)	58.5 (12.4)	158 (79.0)	102 (52.3)	61 (31.3)	32 (16.4)	1.1 (0.7)	3.3 (1.4)	70 (35.0)	n = 110	67 (60.9)	4.4 (18.3)	19.7 (28.6)
Taiwan (n = 313)	53.7 (13.3)	255 (81.5)	132 (42.3)	119 (38.1)	61 (19.6)	0.7 (0.5)	3.8 (1.3)	110 (35.3)	n = 215	104 (48.4)	3.6 (12.5)	31.5 (23.8)
USA (n = 400)	56.2 (13.7)	308 (77.0)	18 (4.9)	132 (35.8)	219 (59.4)	1.0 (0.6)	3.6 (1.4)	183 (45.8)	n = 244	148 (60.7)	7.0 (19.4)	23.4 (24.5)
Venezuela (n = 200)	54.1 (11.3)	181 (90.5)	87 (43.9)	71 (35.9)	40 (20.2)	0.9 (0.6)	3.9 (1.5)	56 (28.0)	n = 144	52 (36.1)	10.4 (28.3)	13.1 (22.0)
UK (n = 43)	59.4 (13.4)	34 (79.1)	0 (0.0)	29 (67.4)	14 (32.6)	1.2 (0.7)	3.7 (1.5)	16 (37.2)	n = 18	10 (55.6)	13.0 (35.2)	20.0 (20.8)
Uruguay (n = 30)	57.7 (10.6)	26 (86.7)	13 (43.3)	13 (43.3)	4 (13.3)	1.0 (0.7)	3.3 (1.9)	12 (40.0)	n = 17	12 (70.6)	4.4 (15.4)	14.2 (29.4)
Total (N = 3920)	*56.3 (13.0)*	*3191 (81.4)*	*1368 (35.6)*	*1530 (39.9)*	*941 (24.5)*	*1.0 (0.7)*	*3.7 (1.6)*	*1225 (31.3)*	*N = 2359*	*1065 (45.22)*	*17.4 (30.5)*	*25.3 (25.6)*

*USA* United States of America, *UK* United Kingdom

Overall 1,065/2359 (45.2%) of persons ≤ 60 years were employed with the highest percentage of employment in Germany (n = 71/113; 62.8%) and the lowest in Morocco (n = 33/181; 18.2%). As only 10 (55.6%) and 12 (70.6%) persons were employed in the UK and Uruguay respectively, these countries were excluded from analyses on absenteeism and presenteeism. Of the 1065 employed persons, 993 completed the question on absenteeism. The percentage of persons in each of the categories for absenteeism was 0% (n = 764 (76.9%)), > 0% and < 100% (n = 164 (16.5%)) and 100% (n = 65 (6.6%)). For the 914 completing the question on presenteeism the distribution was 0% (n = 283 (31.0%)), > 0% and ≤ 30% (n = 420 (46.0%)), > 30% and ≤ 50% (n = 203 (22.2%)) and > 50% and ≤ 100% (n = 8 (0.9%)). The mean absenteeism was 17.4% (SD ± 30.5) and was highest in Morocco 43.8% (SD 43.9) and lowest in Japan 2.6% (SD 9.2%). Among those that could experience presenteeism (employed and not 100% of the time absent), mean presenteeism was 25.3% (SD 25.6) and was highest in Morocco 34.0% (SD 22.3) and lowest in Venezuela 13.1% (SD 22.0). Additional file 2: Table S2 shows characteristics of the employed sample without age restriction.

### Multivariable models with work outcomes

Missing values in covariates were present in 147/2359 (6.2%) cases when employment was the outcome, in 79/993 (8.0%) when absenteeism was the outcome and in 74/914 (8.1%) of those who had completed the presenteeism question (of note: persons with 100% absenteeism could not indicate presenteeism). An overview of missing values per variable in the total sample and differences in characteristics of the sample with and without missing values are presented in the Additional file 3: Table S3 and Additional file 4: Table S4, respectively.

### Employment

Older age (OR 0.98 (95% CI 0.97; 0.99) per year), female gender (OR 0.36 (95% CI 0.28; 0.47)), lower educational level (low vs. high, OR 0.30 (95% CI 0.24; 0.47); medium vs. high, OR 0.63 (95% CI 0.50; 0.78)) were all significantly associated with lower odds of employment. In addition, each point increase in the HAQ score or increase in the DAS28 decreased the odds of employment by 0.64 (95% CI 0.53; 0.76) and 0.83 (95% CI 0.77; 0.89), respectively. However, comorbidities (RDCI) had no significant influence (OR 0.93 (95% CI 0.85; 1.02)).

**Fig. 1:**
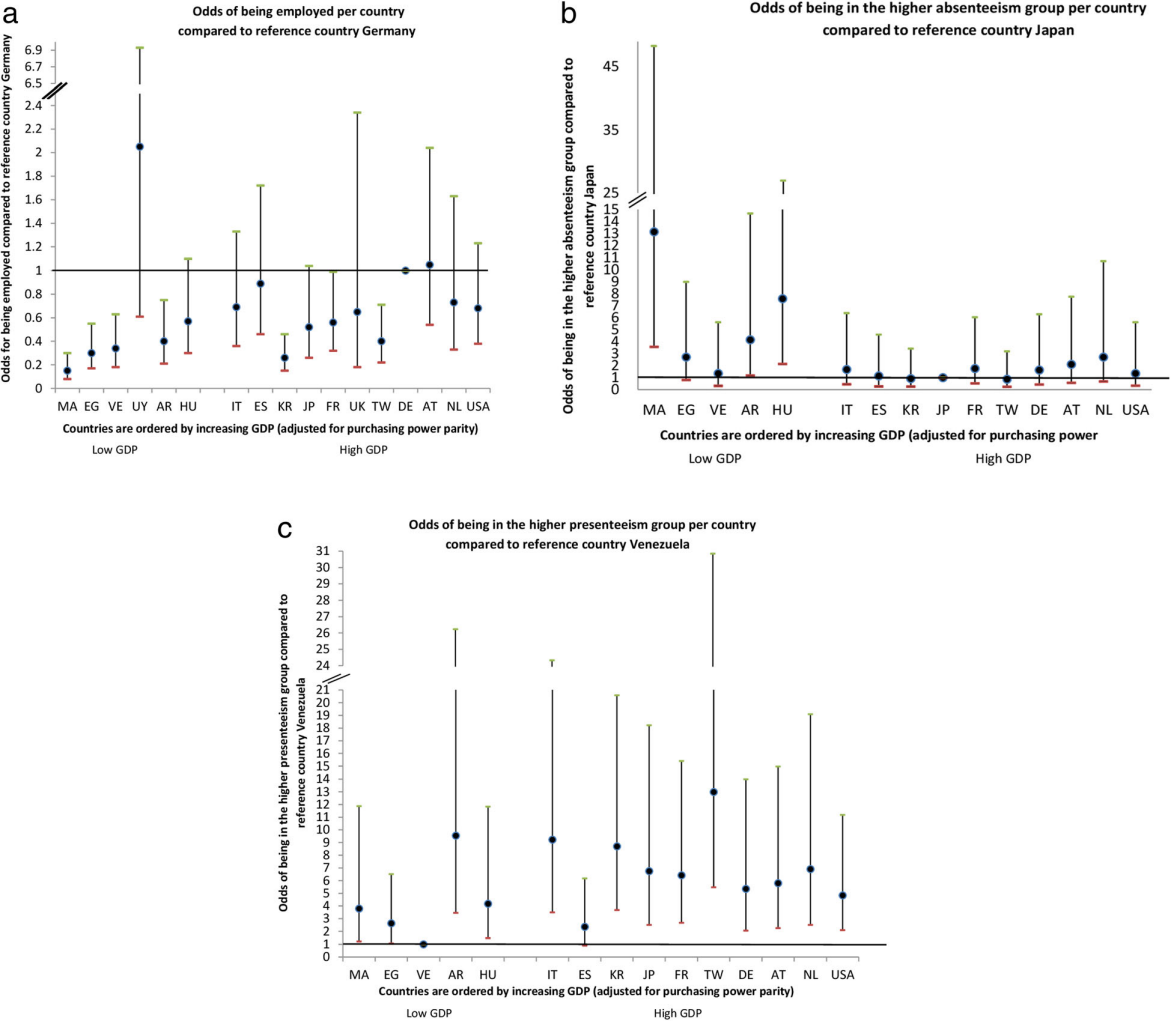
Adjusted association between country of residence and employment, absenteeism and presenteeism using the country with the most favorable work outcome (i.e. highest employment rate and lowest absenteeism and presenteeism) as a reference. **a** Adjusted for age, gender, level of education, modified Health Assessment Questionnaire (mHAQ), 28-joint Disease Activity Score (DAS28) and Rheumatic Disease Comorbidity Index (RDCI). **b** Adjusted for age, gender, mHAQ, DAS28 and RDCI. **c** Adjusted for age, gender, mHAQ and DAS28. MA Morocco, EG Egypt, VE Venezuela, UY Uruguay, AR Argentina, HU Hungary, IT Italy, ES Spain, KR Korea, JP Japan, FR France, UK United Kingdom, TW Taiwan, DE Germany, AT Austria, NL Netherlands, USA United States of America, GDP gross domestic product

In the next step, country of residence was significantly associated with employment. Germany, the country with the highest unadjusted employment was used as the reference category. In Argentina (OR 0.40 (95% CI 0.21; 0.75)), Morocco (OR 0.15 (95% CI 0.08; 0.30)), Egypt (OR 0.30 (95% CI 0.17; 0.55)), Venezuela (OR 0.34 (95% CI 0.18; 0.63)), Korea (OR 0.26 (95% CI 0.15; 0.46)), France (OR 0.56 (95% CI 0.32; 0.99)) and Taiwan (OR 0.40 (95% CI 0.22; 0.71)) there was significantly lower odds of being employed compared to Germany (Fig. 1a).

Patients from low-GDP countries had significantly lower odds of employment compared to high-GDP countries (OR 0.62 (95% CI 0.51; 0.77)), when country was replaced by GDP. As significant interactions were found between GDP and age, gender, level of education and the RDCI, the analyses were stratified by GDP. In high-GDP countries, older age, female gender, higher mHAQ score, higher DAS28 and presence of comorbidities were associated with the chance of not being employed. In lower-GDP countries lower education, higher DAS 28 and female sex reinforced the odds of not being employed (Table 2).

**Table 2:** Association of individual sociodemographic and clinical characteristics with being employed, stratified by gross domestic product (GDP)

	Countries with high GDP, n = 1503 OR (95%CI)	Countries with low GDP, n = 856 OR (95%CI)
Age^a^ (years)	0.97 (0.95; 0.98)	0.99 (0.97; 1.01)
Gender (female vs. male)^a^	0.44 (0.33; 0.60)	0.25 (0.16; 0.41)
Level of education^a^		
Low vs. high	0.50 (0.36; 0.69)	0.18 (0.11; 0.27)
Medium vs. high	0.79 (0.61; 1.02)	0.39 (0.25; 0.59)
mHaq (0–3)	0.53 (0.42; 0.67)	0.94 (0.70; 0.91)
DAS28	0.89 (0.81; 0.97)	0.80 (0.70; 0.91)
Rheumatic Disease Comorbidity Index (0–8)^a^	0.89 (0.81; 0.97)	10.80 (0.70; 0.91)

Results are from multivariable logistic regression analysis. Low GDP-countries: Morocco, Egypt, Venezuela, Uruguay, Argentina, Hungary. High GDP-countries: Italy, Spain, Korea, Japan, France, United Kingdom, Taiwan, Germany, Austria, the Netherlands, United States of America

*Abbreviations*: *OR* odds ratio, *CI* confidence interval, *mHAQ* modified health assessment questionnaire, *DAS28* 28-joint Disease Activity Scale

^a^Significant interaction with GDP (GDP ≤20,000, high; GDP >20,000, low)

Lower HDI and higher SPE, but not higher unemployment, were significantly associated with lower odds of employment and each of the country indices significantly improved the model fit compared to the model without any country indices. Countries in Africa, Latin America and Asia had lower odds of employment compared to Europe. However, there was no significant difference between North America and Europe (Additional file 5: Table S5).

### Absenteeism

The odds of being in a higher category of absenteeism increased with each point increase in the HAQ score (OR 3.08 (95% CI 2.24; 4.23)), the DAS28 (OR 1.33 (95%CI 1.17; 1.51)) and the RDCI (OR 1.46 (95%CI 1.26; 1.69)). When adding country of residence, country was significantly associated with absenteeism. Since Japan had the lowest absenteeism we used it as the reference category.

The odds of being in the next higher category of absenteeism were significantly higher for Argentina (OR 4.17 (95% CI 1.18; 14.67)), Hungary (OR 7.60 (95% CI 2.14; 26.92)) and Morocco (OR 13.15 (95% CI 3.58; 48.29)) compared to Japan (Fig. 1b).

When replacing country of residence by GDP, the odds of being in the higher absenteeism group were higher in low-GDP countries (OR 2.85 (95% CI 1.98; 4.09)) than in high-GDP countries (Table 3). Interactions between GDP and the other covariates were not significant.

**Table 3:** Association of individual sociodemographic and clinical characteristics and gross domestic product (GDP) with absenteeism and presenteeism

	Absenteeism OR (95% CI)	Presenteeism OR (95% CI)
Age (years)	0.99 (0.97; 1.01)	0.99 (0.98; 1.01)
Gender (female vs. male)	0.87 (0.59; 1.29)	0.94 (0.69; 1.29)
mHAQ (0–3)	2.80 (2.02; 3.88)	5.01 (3.60; 6.98)
DAS28	1.25 (1.10; 1.42)	1.63 (1.46; 1.82)
Rheumatic Disease Comorbidity Index (0–8)	1.44 (1.25; 1.68)	-
GDP (low vs. high GDP)	2.85 (1.98; 4.09)	0.49 (0.35; 0.69)

Results of ordinal logistic regression modeling (odds of being in a higher absenteeism or presenteeism group). Absenteeism categories: 1 = 0%; 2 = > 0% to < 100; 3 = 100%. Presenteeism categories: 1 = 0%; 2 = > 0% to 30%; 3 = > 30% to 50%; 4 = > 50–100%

*Abbreviations OR* odds ratio, *CI* confidence interval, *mHAQ* modified Health Assessment Questionnaire, *DAS28* 28-joint Disease Activity Scale

Similarly, lower HDI and higher unemployment rate, but not SPE, were associated with significantly increased odds of being in the higher absenteeism group, and each of the indices significantly improved the model fit compared to the model without any country indices. Countries in Europe, Africa and Latin America had significantly higher odds of absenteeism than countries in Asia (Additional file 5: Table S5).

### Presenteeism

The odds of being in a worse category of presenteeism increased for each point increase in the HAQ score (OR 4.45 (95% CI 3.22; 6.14)) and DAS28 (OR 1.56 (95% CI 1.40; 1.74)). Country of residence was significantly associated with presenteeism. When adding country, Venezuela had the lowest presenteeism and was used as the reference category. All countries except Spain had significantly higher odds of being in a higher presenteeism group compared to Venezuela. The highest odds were for Taiwan (OR 12.99 (95% CI 5.47; 30.85), Korea (OR 8.70 (95% CI 3.68; 20.58)) and Italy (OR 9.23 (95% CI 3.49; 24.31)) (Fig. 1c).

When replacing country of residence by GDP, low-GDP countries had significantly lower odds of being in a higher presenteeism group (0.49 ([0.35; 0.69)) compared to high-GDP countries (Table 3). Interactions between GDP and the other covariates were not significant.

Similarly, lower HDI and higher unemployment rate were associated with lower odds of being in a higher presenteeism group while lower SPE was associated with increased odds of being in the higher presenteeism group. Countries in Asia had significantly higher odds of being in a higher presenteeism group compared to Latin America (Additional file 5: Table S5).

### Contribution of country indices to explanation of the work outcomes

When employment and presenteeism were the outcome, the largest improvement in log likelihood compared to the model without country index was found when the variable HDI was added (log likelihood chi-square test statistic 24.80 and 30.96, respectively; *p* < 0.001). When absenteeism was the outcome, the largest improvement in log likelihood compared to the model without country index was when the variable GDP was added (log likelihood chi-square test statistic 31.34; *p* < 0.001) (Additional file 5: Table S5). Naturally, when adding country of residence or continent the difference was largest for all outcomes due to the number of categories included in this variable, but also indicating the complexity of the construct “country of residence” comprising various cultural and system-related facets.

## Discussion

This study is the first to explore the impact of country of residence on sick leave (absenteeism) and presenteeism (at-work productivity loss) in addition to employment in patients with RA. We showed that country of residence was associated with relevant differences in employment status, absenteeism and presenteeism, independent of individual sociodemographic and clinical characteristics.

Specific-country socioeconomic indices could partially shed light on these differences. As hypothesized, higher economic wealth (GDP) and better human development (HDI) were associated with better employment rates and lower absenteeism among patients with RA. Paradoxically, the direction of association changed when at-work productivity, or presenteeism was the outcome: higher economic wealth and human development and lower unemployment were associated with more presenteeism. When grouping countries by world region, North America had the highest employment and lowest odds of absenteeism. The highest presenteeism was for Asia.

Country differences in work status in persons older than 50 years have been previously investigated in a European comparative household study. Analyses were adjusted for physician-confirmed chronic diseases such as musculoskeletal diseases. The chance of not participating in the labor force among persons with “poor self-perceived health” differed from 1.07 (Sweden) to 3.99 (Switzerland) [24]. As most countries in this study guaranteed access to health care, differences were attributed to availability in regulations for employment protection across countries.

Also in RA, international differences in work status among patients with RA have been reported. Dadoniene et al. assumed better employment with higher economic prosperity, and Mau et al. assumed better employment with lower national rates of economic unemployment [3, 4]. Our findings provided evidence that all mechanisms suggested in the literature might indeed play a role. The HDI, accounting for educational level and life expectancy in addition to GDP, was at least as good and perhaps even a slightly better indicator of employment perspectives. Of course, we can only speculate that higher HDI or GPD is also associated with more efficient support systems for keeping the chronically ill in the labor force. While Sokka suggested that higher work disability among Finish compared to US patients with RA might be attributable to higher income substitution in the case of work disability [6], we failed to show an influence of expenditure on social protection. We need to realize that SPE comprises expenditures for all kind of social hazards. It would be interesting to have a national indicator of the level of income substitution for sick leave or work disability to understand how this influences worker outcomes.

The QUEST-RA study has already revealed differences in employment across a large number of countries, [6] but has not reported the independent magnitude of the effect of GDP. In addition to 1.5-fold higher odds of patients from countries with lower (compared to higher) GDP having no employment, we also showed that women and patients with low education were less likely to be employed. Of interest, in high-GDP countries higher age and more comorbidity had a stronger effect on not being employed. As in QUEST-RA, we saw a clear trend that in low-income countries, patients continue to work with poorer health compared to those in high-income countries, although the interaction was not significant. Overall, these findings suggest age and health restrictions (HAQ, comorbidities) are better acknowledged by the social security system in wealthier countries. Alternatively, increasing job demands and pressure for productivity in wealthier countries make it harder to sustain worker participation at the same level of disease severity in the current economic climate. International combined qualitative and quantitative research might provide more insight into the true explanations.

In the literature we found no studies reporting country differences in sick leave or presenteeism in persons with musculoskeletal disease, and also in RA we are the first to explore the role of country of residence on sick leave and presenteeism. While overall absenteeism followed the same pattern as employment, paradoxically there were higher rates of presenteeism in wealthier countries: likely, patients in high-income countries experience more pressure towards efficiency and productivity. This seems to confirm the suggestion that people in high-income countries experience high work pressure.

Several limitations have to be addressed. First, the national samples might not be representative of patients with RA in participating countries, especially when sample sizes were small. If participation bias was different in low-income compared to high-income countries, likely lower-educated and unemployed patients would have no access to health care in low-income countries and our data would even underestimate the country effect. Second, our study had a cross-sectional design, thus hindering causal inferences. With regard to exposure to country characteristics, it is of note that this factor is present before the outcome. Third, for the classification of countries following different socioeconomic indices the cutoff was chosen based on the distribution of the variable. For some variables (GDP and HDI) there are international classifications but these could not be applied to our sample as the distribution of countries would have been too uneven. For that reason, categories for the indices in the current analyses were based on data inspection that allowed identification of an arbitrary threshold that best discriminated between the two groups. In future research with a larger number of countries, it would be interesting to understand whether there is a specific threshold below which the effect of country wealth or human development adversely influences work outcomes or whether the relationship is more linear. Also, the country indices are not always assessed annually and at a similar point in time in all countries. However, the indices used in this study spanned an acceptable period.

Fourth, in the absence of data on levels of income substitution in the case of disability in sickness absence, we considered SPE as a remote proxy in the case of work restrictions due to illness. It must be noted that the personal insurance for work disability may be a potential confounder for which we were not able to adjust. Fifth, work status in the COMORA study only considered employment (yes/no), and therefore no further analyses could be performed to understand reasons for not participating (anymore) in the labor force. Also, information on presenteeism and absenteeism was collected through self-report from patients and comprised a short time span (7 days). However, it should be noted that the WPAI questionnaire and the recall periods are well-validated Specifically for absenteeism, the short time-frame is valid when the sample is sufficiently large, as sick leave likely occurs randomly between different persons over time [10, 25–27]. Seventh, our models were not adjusted for disease duration, as information in the COMORA study was restricted to identifying those with early disease (≤6 weeks). It is worth noting that in univariable analyses, having early disease was not associated with any of the work outcomes. Moreover in RA, collinearity between age and disease duration is common and often forces to chose on of both factors as confounder. Finally, in order to avoid a dominating impact of differences in the formal age of retirement on the results, the main analyses were restricted to persons age ≤60 years. Notwithstanding, it might also be of interest to compare work outcomes across countries while acknowledging an additional influence of the official retirement age (a well-known country-specific regulation). Additional file 6: Table S6, Additional file 7: Table S7 and Additional file 8: Table S8 and Additional file 9: Figures S1A-C present the results for the total sample (i.e. not excluding those age >60 years). Changes were seen in the raw and adjusted employment rates, but the overall conclusions of the regression models remained unaltered. An expected difference was a stronger impact of age on work status.

This study contributes to the understanding of influence of country of residence on three relevant work outcomes among patients with RA. This is important for international research, as outcomes of interventions aiming to improve health or work ability might not be simply transferrable between countries. Moreover, healthcare providers aiming to improve work outcomes in patients with RA should account for national socioeconomic system characteristics.

## Conclusions

In conclusion, adverse work outcome in patients with rheumatoid arthritis is not only associated with clinical status and individual sociodemographic characteristics but also with the country of residence and country-specific features such as economic wealth, human development or geographical region. Comparison of work outcome across countries should account for system-level contextual factors.

## Additional files

Additional file 1: Table S1. Overview of the countries in the categories of each country index and overlap between categories. (DOCX 1591 kb)

Additional file 2: Table S2. Sociodemographic and clinical characteristics for employed population by country and high and low gross domestic product (GDP). (DOCX 16 kb)

Additional file 3: Table S3. Missing values, *n* (%) in variables by country; *n* (%). (DOCX 15 kb)

Additional file 4: Table S4. Sociodemographic and lifestyle characteristics in subjects with and without missing data in the outcome variables. (DOCX 13 kb)

Additional file 5: Table S5. Overview of odds ratios for each country index (in fully adjusted models) and results of likelihood ratio chi-square (LR chi2) tests comparing models with and without country index variables for the sample age ≤60 years. (DOCX 17 kb)

Additional file 6: Table S6. Overview of odds ratios for each country index (in fully adjusted models) and results of likelihood ratio chi-square (LR chi2) tests comparing models with and without country index variables (for total sample). (DOCX 16 kb)

Additional file 7: Table S7. Association of individual sociodemographic and clinical characteristics with being employed stratified by gross domestic product (GDP) (total sample). (DOCX 14 kb)

Additional file 8: Table S8. Association of individual sociodemographic and clinical characteristics and gross domestic product (GDP) with absenteeism and presenteeism (total sample). (DOCX 13 kb)

Additional file 9: Figure S1A. Odds of being employed compared to reference country (total sample). **Figure S1B.** Odds of being in the higher absenteeism group per country compared to reference country Japan (total sample). **Figure S1C.** Odds of being in the higher presenteeism group per country compared to reference country Venezuela (total sample). (ZIP 77 kb)

## Abbreviations

AR: Argentina
AT: Austria
BMI: Body mass index
COMORA: Comorbidities in Rheumatoid Arthritis
DAS28: 28-Joint Disease Activity Scale
DE: Germany
EG: Egypt
ES: Spain
FR: France
GDP: Gross domestic product
HDI: Human Development Index
HU: Hungary
IT: Italy
JP: Japan
KR: Korea
MA: Morocco
mHAQ: Modified 8-item Health Assessment Questionnaire
NL: The Netherlands
OR: Odds ratio
RA: Rheumatoid arthritis
RDCI: Rheumatic Disease Comorbidity Index
RF: Rheumatoid factor
SD: Standard deviation
SPE: Social protection expenditures
TW: Taiwan
UK: United Kingdom
USA: United States of America
UY: Uruguay
VE: Venezuela
WPAI: Work Productivity Impairment Questionnaire
