# Supplementary material for: Large country differences in work outcomes in patients with RA – an analysis in the multinational study COMORA
Source: Arthritis Res Ther. 2017 Sep 29;19:216. doi: 10.1186/s13075-017-1421-y (PMC5622486; doi:10.1186/s13075-017-1421-y)
Supplement: Supplementary file 1 — Overview of the countries in the categories of each country index and overlap between categories. (DOCX 1591 kb) [file 13075_2017_1421_MOESM1_ESM.docx]

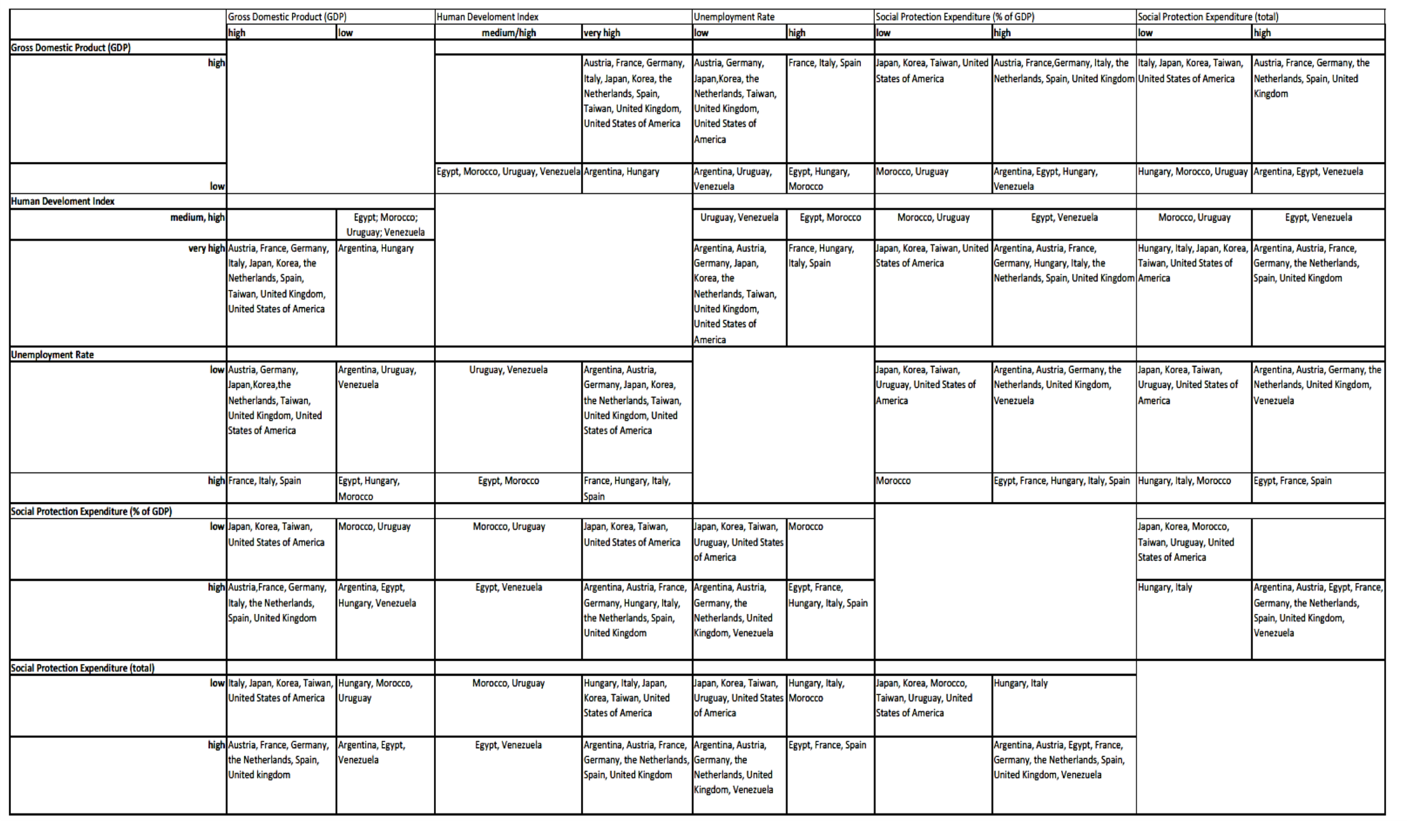


Additional file 1: Table S1 Overview of the countries in the categories of each country index and overlap between categories
