# Supplementary material for: Large country differences in work outcomes in patients with RA – an analysis in the multinational study COMORA
Source: Arthritis Res Ther. 2017 Sep 29;19:216. doi: 10.1186/s13075-017-1421-y (PMC5622486; doi:10.1186/s13075-017-1421-y)
Supplement: Supplementary file 2 — Sociodemographic and clinical characteristics for employed population by country and high and low gross domestic product (GDP). (DOCX 16 kb) [file 13075_2017_1421_MOESM2_ESM.docx]

| Additional file 2: Table S2 Sociodemographic and clinical characteristics for employed population (N=1,225) by country and high and low gross domestic product (GDP) | | | | | | | | | |
| --- | --- | --- | --- | --- | --- | --- | --- | --- | --- |
|  | **Age,  mean (SD)** | **Women, n (%)** | **Level of education, n (%)** | | | **mHAQ,  mean (SD)** | **Das28,  mean (SD)** | **Percent absenteeism, mean (SD)** | **Percent presenteeism,  mean (SD)** |
|  | | | **Low** | **Medium** | **High** |  | | | |
| Austria, (n=56) | 45.8 (9.1) | 45 (72.6) | 33 (58.9) | 16 (28.6) | 13 (23.2) | 0.9 (0.6) | 3.1 (1.4) | 9.2 (24.5) | 26.2 (23.4) |
| Argentina, (n=62) | 51.9 (9.5) | 45 (80.4) | 17 (27.4) | 27 (43.5) | 12 (19.4) | 1.0 (0.6) | 3.6 (1.4) | 20.6 (35.3) | 31.9 (31.2) |
| Egypt, (n=76) | 45.1 (10.4) | 46 (60.5) | 26 (34.2) | 16 (21.1) | 34 (44.7) | 1.3 (0.7) | 5.1 (1.7) | 26.8 (33.7) | 31.7 (25.9) |
| France, (n=132) | 49.8 (9.4) | 106 (80.3) | 17 (12.9) | 63 (47.7) | 52 (39.4) | 0.7 (0.4) | 2.7 (1.4) | 8.6 (25.6) | 20.2 (22.2) |
| Germany, (n=74) | 49.7 (7.4) | 56 (75.7) | 0 (0.00) | 61 (82.4) | 13 (17.6) | 0.8 (0.5) | 3.2 (1.4) | 10.8 (29.8) | 26.6 (25.1) |
| Hungary, (n=50) | 48.1 (11.4) | 42 (84.0) | 8 (16.0) | 24 (48.0) | 18 (36.0) | 1.0 (0.6) | 3.2 (1.3) | 35.5 (46.2) | 21.8 (24.0) |
| Italy, (n=56) | 49.9 (9.7) | 39 (69.6) | 18 (32.1) | 29 (51.8) | 9 (16.1) | 0.8 (0.5) | 3.6 (1.8) | 11.2 (27.1) | 31.4 (29.8) |
| Japan, (n=71) | 56.5 (12.3) | 49 (69.0) | 11 (15.5) | 49 (69.0) | 11 (15.5) | 0.8 (0.5) | 2.8 (1.4) | 3.0 (9.6) | 20.4 (22.5) |
| Korea, (n=118) | 50.9 (10.4) | 85 (72.7) | 19 (16.1) | 56 (47.5) | 43 (36.4) | 0.7 (0.4) | 3.3 (1.3) | 4.7 (16.2) | 26.9 (25.0) |
| Morocco, (n=34) | 47.5 (8.4) | 24 (70.6) | 8 (23.5) | 10 (29.4) | 16 (47.1) | 1.0 (0.7) | 4.4 (1.3) | 45.6 (44.4) | 34.0 (22.3) |
| Netherlands, (n=49) | 48.6 (9.5) | 30 (61.2) | 0 (0.00) | 4 (8.2) | 41 (83.7) | 1.0 (0.6) | 2.4 (1.2) | 15.2 (31.7) | 28.3 (32.3) |
| Spain, (n=70) | 48.9 (7.4) | 58 (82.9) | 20 (28.6) | 31 (44.3) | 18 (25.7) | 0.8 (0.5) | 2.9 (1.4) | 5.8 (21.6) | 20.2 (28.3) |
| Taiwan, (n=110) | 45.5 (10.1) | 79 (71.8) | 20 (18.2) | 61 (55.5) | 29 (26.4) | 0.6 (0.4) | 3.8 (1.3) | 3.4 (12.1) | 31.2 (23.7) |
| USA, (n=183) | 50.3 (11.9) | 131 (71.6) | 7 (3.8) | 47 (25.7) | 115 (62.8) | 0.8 (0.5) | 3.2 (1.3) | 7.2 (19.6) | 23.7 (24.8) |
| Venezuela, (n=56) | 47.1 (10.9) | 47 (83.9) | 16 (28.6) | 19 (33.9) | 21 (37.5) | 0.7 (0.5) | 3.4 (1.4) | 9.4 (27.1) | 11.8 (21.2) |
| Gross domestic product (GDP) * | | | | | | | | | |
| High GDP, (n=925) | 49.6(10.4) | 678 (73.3) | 145 (15.7) | 417 (45.1) | 344 (37.2) | 0.8 (0.5) | 3.1 (1.4) | 7.2 (21.7) | 25.2 (25.3) |
| Low GDP, (n=272) | 47.8 (10.5) | 204 (75.0) | 75 (27.6) | 96 (35.3) | 101 (37.1) | 1.0 (0.6) | 4.0 (1.6) | 26.5 (38.5) | 26.6 (26.8) |
| * *Countries in* ***high GDP-group****: AT; FR; DE; IT; JP; KR; NL; ES; TW; USA Countries in* ***low GDP-group****: AR; EG; HU; MA; VE* | | | | | | | | | |
