# Supplementary material for: Large country differences in work outcomes in patients with RA – an analysis in the multinational study COMORA
Source: Arthritis Res Ther. 2017 Sep 29;19:216. doi: 10.1186/s13075-017-1421-y (PMC5622486; doi:10.1186/s13075-017-1421-y)
Supplement: Supplementary file 3 — Missing values, n (%) in variables by country; n (%). (DOCX 15 kb) [file 13075_2017_1421_MOESM3_ESM.docx]

| Additional file 3: Table S3 Missing values n (%) in variables by country; n (%) | | | | | | | | | | | |
| --- | --- | --- | --- | --- | --- | --- | --- | --- | --- | --- | --- |
|  | | | | | | | | | | **Employed sample only** | |
|  | **Age** | **Gender** | **Level of education** | **mHAQ** | **DAS28** | **GDP** | **Un- employment rate** | **Rheumatic disease comorbidity index** | **Employed (y/n)** | **Percent  absenteeism** | **Percent presenteeism** |
| **Argentina n=200** | - | - | - | - | 7 (3.5) | - | - | - | - | 2 (3.6) | 9 (16.1) |
| **Austria n=204** | - | - | - | 1 (0.5) | 3 (1.5) | - | - | - | - | 6 (9.7) | 10 (16.1) |
| **Egypt n=308** | - | - | - | - | 2 (0.7) | - | - | - | - | - | 6 (7.9) |
| **France n=411** | - | 2 (0.5) | 2 (0.5) | 14 (3.4) | 13 (3.2) | - | - | - | 6 (1.5) | 10 (7.6) | 31 (23.5) |
| **Germany n=209** | - | - | - | 1 (0.5) | 75 (35.9) | - | - | - | - | 4 (5.4) | 11 (14.9) |
| **Hungary n=201** | - | - | - | - | - | - | - | - | - | - | 17 (34.0) |
| **Italy n=228** | - | - | - | - | - | - | - | - | - | 4 (7.1) | 7 (12.5) |
| **Japan n=207** | - | - | - | - | 6 (2.9) | - | - | - | - | - | - |
| **Korea n=400** | - | 1 (0.3) | 1 (0.3) | - | 1 (0.3) | - | - | - | 1 (0.3) | 2 (1.7) | 5 (4.2) |
| **Morocco  n=227** | - | 6 (2.6) | 6 (2.6) | 10 (4.4) | 15 (6.6) | - | - | - | 10 (4.4) | 4 (11.8) | 14 (41.2) |
| **Netherlands  n=139** | - | - | 33 (23.7) | 11 (7.9) | 8 (5.8) | - | - | - | 2 (1.4) | 4 (8.2) | 7 (14.3) |
| **Spain  n=200** | - | 1 (0.5) | 5 (2.5) | 11 (5.5) | 4 (2.0) | - | - | - | - | 8 (11.4) | 10 (14.3) |
| **Taiwan  n=313** | - | 1 (0.3) | 1 (0.3) | 1 (0.3) | 3 (1.0) | - | - | - | 1 (0.3) | 5 (4.6) | 6 (5.5) |
| **USA**  **n=400** | - | - | 31 (7.8) | 8 (2.0) | 38 (9.5) | - | - | - | 2 (0.5) | 10 (5.5) | 17 (9.3) |
| **Venezuela**  **n=200** | - | - | 2 (1.0) | 1 (0.5) | 11 (5.5) | - | - | - | 1 (0.5) | 14 (25.0) | 16 (28.6) |
