# Supplementary material for: Large country differences in work outcomes in patients with RA – an analysis in the multinational study COMORA
Source: Arthritis Res Ther. 2017 Sep 29;19:216. doi: 10.1186/s13075-017-1421-y (PMC5622486; doi:10.1186/s13075-017-1421-y)
Supplement: Supplementary file 4 — Sociodemographic and lifestyle characteristics in subjects with and without missing data in the outcome variables. (DOCX 13 kb) [file 13075_2017_1421_MOESM4_ESM.docx]

| Additional file 4: Table S4 Socio-demographic and lifestyle characteristics in subjects with and without missing data in the outcome variables | | | | | | |
| --- | --- | --- | --- | --- | --- | --- |
| Outcome variables | Employment | | Absenteeism | | Presenteeism | |
|  | No missing values | Missing values | No missing values | Missing values | No missing values | Missing values |
| Age, mean (SD) | 56.2 (13.0) | 63.3 (13.9) | 49.2 (10.5) | 49.8 (10.2) | 49.2 (10.5) | 49.5 (10.2) |
| Women, n (%) | 3180 (81.6) | 11 (91.7) | 835 (73.4) | 68 (79.1) | 770 (73.2) | 133 (77.3) |
| Level of education, n (%) |  |  |  |  |  |  |
| Low | 1364 (35.6) | 4 (36.4) | 211 (18.8) | 14 (16.5) | 193 (18.6) | 32 (18.9) |
| Medium | 1526 (39.9) | 4 (36.4) | 483 (43.1) | 43 (50.6) | 447 (43.1) | 79 (46.8) |
| High | 938 (24.5) | 3 (27.3) | 427 (38.1) | 28 (32.9) | 397 (38.3) | 58 (34.3) |
| mHAQ, mean (SD) | 1.0 (0.7) | 1.1 (0.5) | 0.8 (0.5) | 0.7 (0.4) | 0.8 (0.5) | 0.9 (0.6) |
| DAS28, mean (SD)) | 3.7 (1.6) | 3.6 (0.5) | 3.3 (1.5) | 3.2 (1.2) | 3.3 (1.5) | 3.6 (1.5) |
| Rheumatic disease comorbidity index, mean (SD) | 1.2 (1.2) | 0.4 (0.8) | 0.9 (1.1) | 0.6 (1.0) | 0.9 (1.1) | 0.9 (1.2) |
