# Supplementary material for: Large country differences in work outcomes in patients with RA – an analysis in the multinational study COMORA
Source: Arthritis Res Ther. 2017 Sep 29;19:216. doi: 10.1186/s13075-017-1421-y (PMC5622486; doi:10.1186/s13075-017-1421-y)
Supplement: Supplementary file 5 — Overview of odds ratios for each country index (in fully adjusted models) and results of likelihood ratio chi-square (LR chi2) tests comparing models with and without country index variables for the sample age ≤60 years. (DOCX 17 kb) [file 13075_2017_1421_MOESM5_ESM.docx]

| Additional file 5: Table S5 Overview of odds ratios for each country index (in fully adjusted models) and results of likelihood ratio chi-square (LR chi^2^) tests comparing models with and without country index variables | | | | |
| --- | --- | --- | --- | --- |
|  | OR [95%CI] | Log-likelihood | LR chi^2^ test^Ϯ^ | p-value for LR chi^2^ test |
| **Outcome “employment”** | | | | |
| no index | - | -1332.20 |  | |
| GDP (reference: high) | 0.62 [0.51;0.77] | -1322.11 | 20.18 | <0.001 |
| HDI (reference: high) | 0.55 [0.43;0.70] | -1319.80 | 24.80 | <0.001 |
| SPE (international dollars; reference: high) | 0.70 [0.57;0.84] | -1325.30 | 13.79 | <0.001 |
| SPE (percentage of GDP; reference: high) | 0.78[0.65;0.94] | -1328.85 | 6.69 | 0.01 |
| Unemployment rate (reference: low) | 0.91 [0.75;1.11] | -1331.77 | 0.85 | 0.36 |
| Continent  (reference: North America) |  | -1297.56 | 69.35 | <0.001 |
| Africa | 0.32 [0.24;0.44] |  | | |
| Latin America | 0.56 [0.41;0.76] |  |  |  |
| North America | 0.95 [0.66;1.36] |  |  |  |
| Asia | 0.48 [0.37;0.61] |  |  |  |
| **Outcome “absenteeism”*** | | | | |
| no index | - | -526.70 |  | |
| GDP (reference: high) | 2.84 [1.98;4.09] | -511.03 | 31.34 | <0.001 |
| HDI (reference: high) | 1.97 [1.30;2.30] | -521.84 | 9.73 | 0.002 |
| SPE (international dollars; reference: high) | 0.73 [0.51;1.04] | -525.18 | 3.06 | 0.08 |
| SPE (percentage of GDP; reference: high) | 0.97 [0.69;1.37] | -526.69 | 0.03 | 0.87 |
| Unemployment rate (reference: low) | 1.82 [1.29;2.55] | -520.85 | 11.70 | <0.001 |
| Continent  (reference: Asia) |  | -512.88 | 27.65 | <0.001 |
| Europe | 2.28 [1.32;3.92] |  | | |
| Africa | 4.63 [2.51;8.55] |  |  |  |
| Latin America | 2.86 [1.43;5.69] |  |  |  |
| North America | 1.74 [0.88;3.43] |  |  |  |
| **Outcome “presenteeism”*** | | | | |
| no index | - | -780.23 |  | |
| GDP (reference: high) | 0.49 [0.35;0.69] | -771.70 | 17.06 | <0.001 |
| HDI (reference: high) | 0.31 [0.21;0.48] | -764.75 | 30.96 | <0.001 |
| SPE (international dollars; reference: high) | 1.57 [1.19;2.07] | -775.03 | 10.40 | 0.001 |
| SPE (percentage of GDP; reference: high) | 1.65 [1.25;2.17] | -773.76 | 12.93 | <0.001 |
| Unemployment rate (reference: low) | 0.69 [0.52;0.91] | -776.86 | 6.74 | 0.01 |
| Continent  (reference: Latin America) |  | -764.94 | 30.57 | <0.001 |
| Europe | 1.64 [0.97;2.78] |  | | |
| Africa | 0.85 [0.45;1.63] |  |  |  |
| North America | 1.45 [0.80;2.63] |  |  |  |
| Asia | 2.92 [1.69;5.04] |  |  |  |
| *^Ϯ^ Minus two (i.e. -2) times the difference between the log likelihood of the model with and without the country index)* **Results of ordinal logistic regression models (odds of being in a higher absenteeism or presenteeism group);*  *absenteeism categories: 1=0%; 2=>0% to <100; 3=100%, presenteeism categories: 1= 0%; 2=>0% to 30%; 3=>30% to 50% ; 4=>50-100% ,* GDP= Gross domestic product; HDI= Human development index; SPE= Social protection expenditure | | | | |
