# Supplementary material for: Large country differences in work outcomes in patients with RA – an analysis in the multinational study COMORA
Source: Arthritis Res Ther. 2017 Sep 29;19:216. doi: 10.1186/s13075-017-1421-y (PMC5622486; doi:10.1186/s13075-017-1421-y)
Supplement: Supplementary file 6 — Overview of odds ratios for each country index (in fully adjusted models) and results of likelihood ratio chi-square (LR chi2) tests comparing models with and without country index variables (for total sample). (DOCX 16 kb) [file 13075_2017_1421_MOESM6_ESM.docx]

| Additional file 6: Table S6 Overview of odds ratios for each country index (in fully adjusted models) and results of likelihood ratio chi-square (LR chi^2^) tests comparing models with and without country index variables  (for total sample, N=3920) | | | | |
| --- | --- | --- | --- | --- |
|  | OR [95%CI] | Log-likelihood | LR chi^2^ test^Ϯ^ | p-value for LR chi^2^ test |
| **Outcome “employment”** | | | | |
| no index | - | -1794.94 |  | |
| GDP (reference: high) | 0.66 [0.54;0.80] | -1785.96 | 17.96 | <0.001 |
| HDI (reference: high) | 0.55 [0.43;0.69] | -1776.00 | 26.10 | <0.001 |
| SPE (international dollars; reference: high) | 0.91 [0.77;1.07] | -1794.28 | 1.32 | 0.25 |
| SPE (percentage of GDP; reference: high) | 0.88 [0.74;1.03] | -1793.70 | 2.48 | 0.12 |
| Unemployment rate (reference: low) | 0.79 [0.66;0.93] | -1791.09 | 7.70 | 0.01 |
| Continent  (reference: North America) |  | -1769.04 | 51.79 | <0.001 |
| Europe | 0.78 [0.58;1.04] |  | | |
| Africa | 0.29 [0.20;0.43] |  |  |  |
| Latin America | 0.63 [0.44;0.91] |  |  |  |
| Asia | 0.58 [0.42;0.79] |  |  |  |
| **Outcome “absenteeism”*** | | | | |
| no index | - | -614.02 |  | |
| GDP (reference: high) | 2.68 [1.91;3.76] | -598.28 | 31.47 | <0.001 |
| HDI (reference: high) | 1.95 [1.30;2.92] | -608.95 | 10.15 | 0.001 |
| SPE (international dollars; reference: high) | 0.95 [0.69;1.30] | -613.97 | 0.11 | 0.74 |
| SPE (percentage of GDP; reference: high) | 0.72 [0.52;0.99] | -611.97 | 4.11 | 0.04 |
| Unemployment rate (reference: low) | 1.87 [1.36;2.56] | -606.67 | 14.70 | <0.001 |
| Continent  (reference: Asia) |  | -599.90 | 28.23 | <0.001 |
| Europe | 1.98 [1.25;3.13] |  | | |
| Africa | 4.04 [2.33;7.03] |  |  |  |
| Latin America | 2.21 [1.20;4.08] |  |  |  |
| North America | 1.32 [0.73;2.39] |  |  |  |
| **Outcome “presenteeism”*** | | | | |
| no index | - | -904.51 |  | |
| GDP (reference: high) | 0.45 [0.32;0.62] | -892.38 | 24.27 | <0.001 |
| HDI (reference: high) | 0.31 [0.21;0.46] | -886.99 | 35.06 | <0.001 |
| SPE (international dollars; reference: high) | 1.64 [1.28;2.10] | -897.47 | 14.09 | <0.001 |
| SPE (percentage of GDP; reference: high) | 1.60 [1.24;2.07] | -897.87 | 13.29 | <0.001 |
| Unemployment rate (reference: low) | 0.70 [0.54;0.92] | -901.16 | 6.70 | 0.01 |
| Continent  (reference: Latin America) |  | -886.48 | 36.06 | <0.001 |
| Europe | 1.94 [1.19;3.16] |  | | |
| Africa | 0.99 [0.54;1.85] |  |  |  |
| North America | 1.80 [1.05;3.11] |  |  |  |
| Asia | 3.27 [1.98;5.42] |  |  |  |
| *^Ϯ^ Minus two (i.e. -2) times the difference between the log likelihood of the model with and without the country index)* **Results of ordinal logistic regression models (odds of being in a higher absenteeism or presenteeism group);*  *absenteeism categories: 1=0%; 2=>0% to <100; 3=100%, presenteeism categories: 1= 0%; 2=>0% to 30%; 3=>30% to 50% ; 4=>50-100% ,* GDP= Gross domestic product; HDI= Human development index; SPE= Social protection expenditure | | | | |
