# Supplementary material for: Large country differences in work outcomes in patients with RA – an analysis in the multinational study COMORA
Source: Arthritis Res Ther. 2017 Sep 29;19:216. doi: 10.1186/s13075-017-1421-y (PMC5622486; doi:10.1186/s13075-017-1421-y)
Supplement: Supplementary file 7 — Association of individual sociodemographic and clinical characteristics with being employed stratified by gross domestic product (GDP) (total sample). (DOCX 14 kb) [file 13075_2017_1421_MOESM7_ESM.docx]

| Additional file 7: Table S7 Association of individual sociodemographic & clinical characteristics with being employed stratified by gross domestic product (GDP)***** | | |
| --- | --- | --- |
|  | **Countries with high GDP;**  **n=2,754**  **OR [95%CI]** | **Countries with low GDP;**  **n=1,166**  **OR [95%CI]** |
| Age^1^ (years) | 0.92 [0.91;0.93] | 0.96 [0.94;0.97] |
| Gender (female vs. male)^1^ | 0.44 [0.35;0.56] | 0.24 [0.16;0.36] |
| Level of education^1^ | | |
| Low vs. high | 0.44 [0.34;0.58] | 0.23 [0.16;0.35] |
| Medium vs. high | 0.79 [0.63;0.99] | 0.42 [0.28;0.62] |
| mHaq (0-3) | 0.55 [0.45;0.67] | 0.83 [0.63;1.10] |
| DAS28 | 0.90 [0.82;0.98] | 0.79 [0.70;0.89] |
| Rheumatic Disease Comorbidity Index ^1^(0-8) | 0.90 [0.84;0.98] | 1.02 [0.88;1.18] |
| **Results from multivariable logistic regression analysis ^1^ Significant interaction with GDP (GDP≤ 20,000: high; GDP > 20,000: low) Abbreviations: OR: odds ratio; CI: confidence interval; mHAQ: modified health assessment questionnaire; DAS28: 28-joints disease activity scale*  *High GDP-countries: Morocco, Egypt, Venezuela, Uruguay, Argentina, Hungary Low GDP-countries: Italy, Spain, Korea, Japan, France, United Kingdom, Taiwan, Germany, Austria, the Netherlands, United States of America* | | |
