# Supplementary material for: Large country differences in work outcomes in patients with RA – an analysis in the multinational study COMORA
Source: Arthritis Res Ther. 2017 Sep 29;19:216. doi: 10.1186/s13075-017-1421-y (PMC5622486; doi:10.1186/s13075-017-1421-y)
Supplement: Supplementary file 8 — Association of individual sociodemographic and clinical characteristics and gross domestic product (GDP) with absenteeism and presenteeism (total sample). (DOCX 13 kb) [file 13075_2017_1421_MOESM8_ESM.docx]

| Additional file 8: Table S8   Association of individual sociodemographic & clinical characteristics and gross domestic product (GDP) with absenteeism and presenteeism | | |
| --- | --- | --- |
|  | **Absenteeism**  **OR [95% CI]** | **Presenteeism**  **OR [95% CI]** |
| Age (years) | 0.99 [0.98;1.02] | 0.99 [0.98;1.01] |
| Gender (female vs male) | 0.82 [0.58;1.17] | 0.90 [0.68;1.20] |
| mHAQ (0-3) | 2.68 [1.98;3.63] | 4.85 [3.57;6.61] |
| DAS28 | 1.26 [1.12;1.42] | 1.64 [1.48;1.82] |
| Rheumatic Disease Comorbidity Index (0-8) | 1.39 [1.21;1.59] | - |
| GDP (low vs high GDP) | 2.68 [1.91;3.76] | 0.45 [0.32;0.62] |
| *Results of ordinal logistic regression models (odds of being in a higher absenteeism or presenteeism group)*  *absenteeism categories: 1=0%; 2=>0% to <100; 3=100%*  *presenteeism categories: 1= 0%; 2=>0% to 30%; 3=>30% to 50% ; 4=>50-100%  abbreviations: OR: odds ratio; CI: confidence interval; mHAQ: modified health assessment questionnaire; DAS28: 28-joints disease activity scale* | | |
