# Supplementary figures and images for: Large country differences in work outcomes in patients with RA – an analysis in the multinational study COMORA
Source: Arthritis Res Ther. 2017 Sep 29;19:216. doi: 10.1186/s13075-017-1421-y (PMC5622486; doi:10.1186/s13075-017-1421-y)

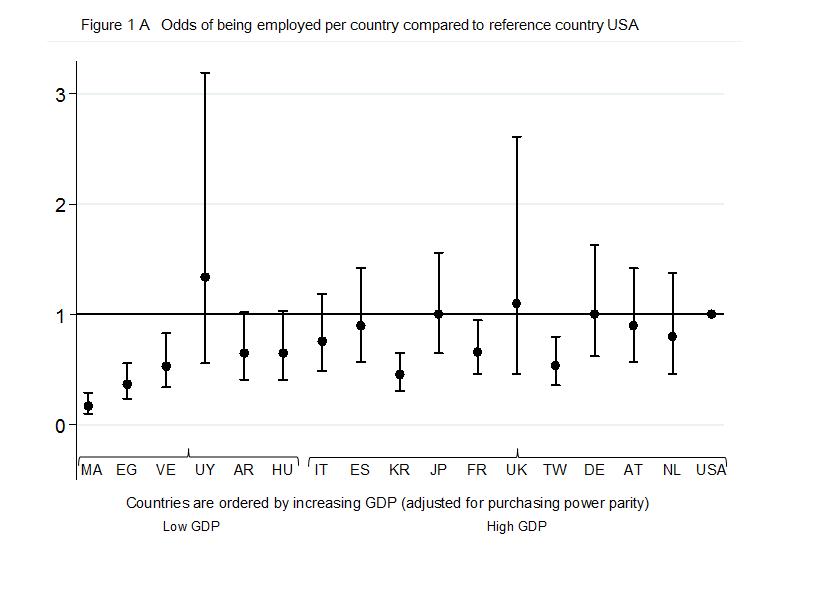

Supplement: Supplementary file 9 — Odds of being employed compared to reference country (total sample). Figure S1B. Odds of being in the higher absenteeism group per country compared to reference country Japan (total sample). Figure S1C. Odds of being in the higher presenteeism group per country compared to reference country Venezuela (total sample). (ZIP 77 kb) [file 13075_2017_1421_MOESM9_ESM.zip › supplementary figure 1A.docx]

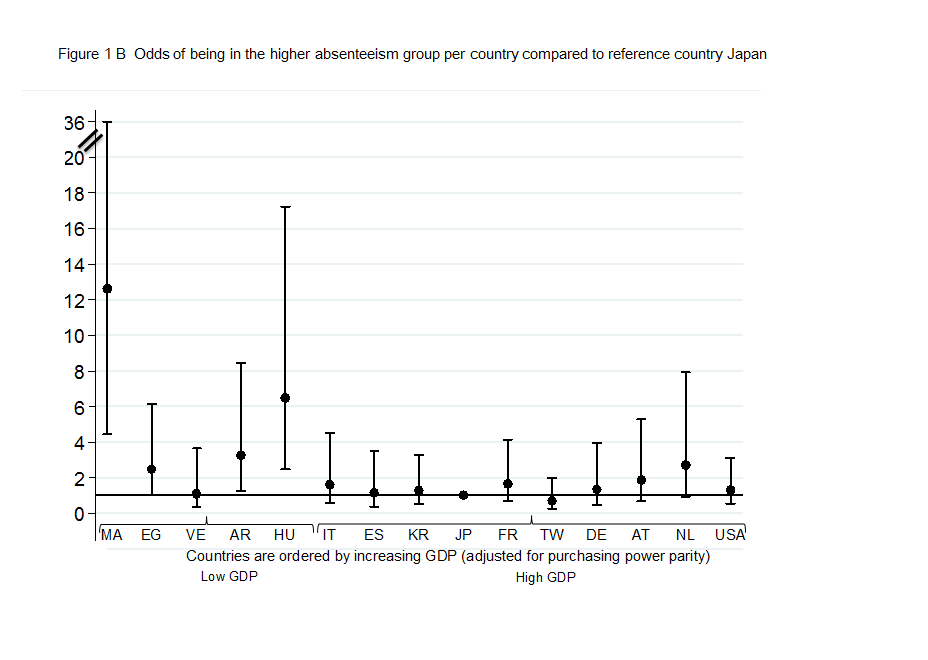

Supplement: Supplementary file 9 — Odds of being employed compared to reference country (total sample). Figure S1B. Odds of being in the higher absenteeism group per country compared to reference country Japan (total sample). Figure S1C. Odds of being in the higher presenteeism group per country compared to reference country Venezuela (total sample). (ZIP 77 kb) [file 13075_2017_1421_MOESM9_ESM.zip › supplementary figure 1b.docx]

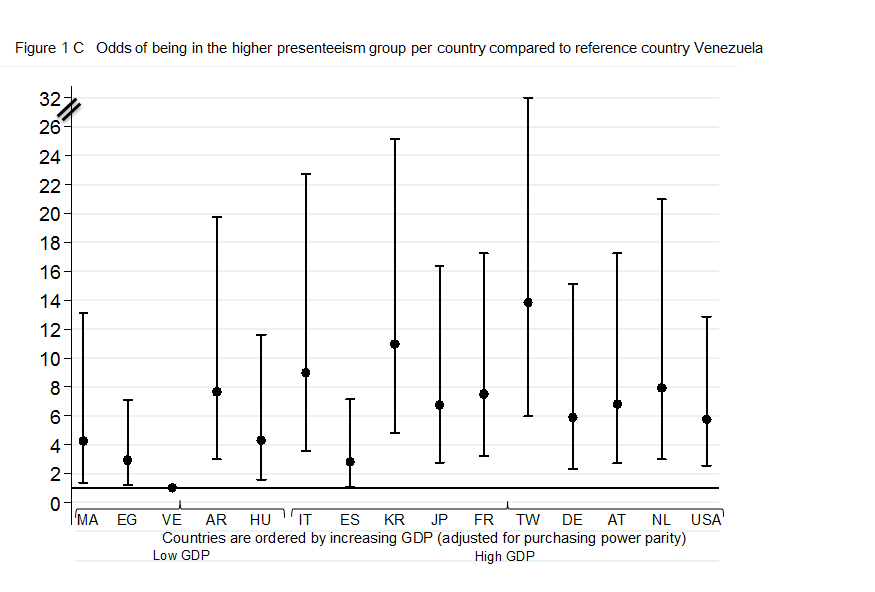

Supplement: Supplementary file 9 — Odds of being employed compared to reference country (total sample). Figure S1B. Odds of being in the higher absenteeism group per country compared to reference country Japan (total sample). Figure S1C. Odds of being in the higher presenteeism group per country compared to reference country Venezuela (total sample). (ZIP 77 kb) [file 13075_2017_1421_MOESM9_ESM.zip › supplementary figure 1c.docx]
